# *EDDIDAT*: a graphical user interface for the analysis of energy-dispersive diffraction data

**DOI:** 10.1107/S1600576720005506

**Published:** 2020-06-12

**Authors:** Daniel Apel, Martin Genzel, Matthias Meixner, Mirko Boin, Manuela Klaus, Christoph Genzel

**Affiliations:** a Helmholtz-Zentrum-Berlin für Materialien und Energie, Berlin, Germany; b Technische Universität Berlin, Institut für Mathematik, Berlin, Germany

**Keywords:** energy-dispersive X-ray diffraction, depth-resolved residual stress analysis, X-ray diffraction data software

## Abstract

*EDDIDAT* is a MATLAB-based graphical user interface for the convenient and versatile analysis of energy-dispersive diffraction data obtained at laboratory and synchrotron sources. The main focus of *EDDIDAT* up to now has been on the analysis of residual stresses, but it can also be used to prepare measurement data for subsequent phase analysis or analysis of preferred orientation. The program provides access to the depth-resolved analysis of residual stresses at different levels of approximation. Furthermore, the graphic representation of the results also serves for the consideration of microstructural and texture-related properties. The included material database allows for the quick analysis of the most common materials and is easily extendable. The plots and results produced with *EDDIDAT* can be exported to graphics and text files. *EDDIDAT* is designed to analyze diffraction data from various energy-dispersive X-ray sources. Hence it is possible to add new sources and implement the device-specific properties into *EDDIDAT*. The program is freely available to academic users.

## Abstract 2

*EDDIDAT* is a program that provides a graphical user interface for the evaluation of energy-dispersive X-ray diffraction data with the focus on depth-resolved residual stress analysis.

## 1. Introduction   

The energy-dispersive diffraction (EDD) method is a powerful tool in many fields of materials research such as residual stress, texture and crystal structure analysis. The main advantage of the EDD method is the little effort needed compared with angle-dispersive diffraction (ADD). The EDD method involves a simple and fixed instrumental setup that allows for measuring complete diffraction patterns with a multitude of diffraction lines *E* ^*hkl*^ in a nondestructive fashion. As a consequence, large data sets are collected in a short time, which makes it necessary to provide users with software that is reliable and easy to handle in order to process all recorded diffraction spectra in a reasonable time. It is often challenging for laboratory researchers and manufacturers to gain access to high-energy synchrotron X-ray experimental stations since the availability of the EDD method and EDD beamlines is limited. Currently, there are only a few beamlines that provide white-beam diffraction in an energy range suitable for conducting diffraction experiments, especially with regard to residual stress analysis. These include the ID15 beamline at the ESRF, the BL28B2 beamline at SPring-8, the PSICHÉ beamline at SOLEIL and the I12 beamline at Diamond Light Source. The beamline P61A PETRA III is currently in commissioning. However, Genzel *et al.* (2011 ▸) showed that it is possible to modify a standard angle-dispersive diffractometer with relatively little effort to be used in the energy-dispersive (ED) mode of diffraction, allowing one to benefit from the advantages of the EDD method on a laboratory scale. Apel *et al.* (2018 ▸ *a*, 2018 ▸ *b*) introduced a new type of diffractometer that exploits the features of the EDD method and also further enhances its applicability by creating new measurement methods. The development of new X-ray sources like the liquid-metal jet X-ray source (LMJ) makes it possible to further reduce the gap in performance regarding the photon flux of ‘laboratory beamlines’ compared with synchrotron beamlines. Consequently, this further enhances the possibilities to downscale the EDD method to laboratory conditions and therefore increase the opportunities for performing EDD experiments. Wansleben *et al.* (2019 ▸) recently reported absolute photon-flux measurements at a 70 kV MetalJet source, which confirm corresponding simulation calculations and thus prove the performance of these facilities. At the X-ray CoreLab of the Helmholtz-Zentrum Berlin (HZB), three laboratory EDD instruments, two of them operated with an LMJ source and the other with a conventional tungsten X-ray tube (LIMAX-70, LIMAX-160 and LEDDI), are available for users. Because of the rather low dissemination of the EDD method, the availabilty of software suites to handle such data is limited [for example one could use *GSAS* (Larson & Von Dreele, 2000 ▸) and *TOPAS* (Bruker, 2003 ▸; Coelho, 2018 ▸), but these programs were primarily developed for the evaluation of ADD data]. With the MATLAB-based (The MathWorks Inc., Natick, Massachusetts, USA) EDD analysis tool *EDDIDAT* (energy-dispersive diffraction data analysis tool) the authors present a data analysis tool that was developed strictly for the analysis of EDD data. The current version of *EDDIDAT* focuses on the evaluation of EDD data with regard to the analysis of residual stresses. However, it can also be used, for example, to prepare measurement data for subsequent phase analysis or analysis of preferred orientation. It is available to academic users upon request to the authors at HZB.

## 2. Theory of ED depth-resolved X-ray stress analysis   

For detailed information about X-ray stress analysis (XSA) the authors refer to the available textbooks (for example, Hauk, 1997 ▸; Noyan & Cohen, 1987 ▸; Spiess *et al.*, 2019 ▸; Birkholz, 2006 ▸). Here, only a brief summary is presented in order to explain the basis of the residual stress analysis used in *EDDIDAT*.

The basic equation of ED diffraction is given by (Giessen & Gordon, 1968 ▸)
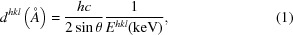
where *d* ^*hkl*^ is the lattice spacing, θ is the Bragg angle, *h* is the Planck constant, *c* is the speed of light and *E* ^*hkl*^ is the energy of the diffraction line *hkl*. As mentioned before, in the EDD mode, complete diffraction spectra that contain a multitude of diffraction lines are recorded. Each diffraction line *E* ^*hkl*^ can be assigned to a specific average information depth τ^*hkl*^, which generally can be expressed by
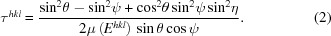
μ(*E* ^*hkl*^) is the energy-dependent linear absorption coefficient, and ψ and η denote the tilt angle and the sample rotation around the diffraction vector, respectively. Hence, the EDD method is particularly suitable for the depth-resolved analysis of residual stresses. Since residual stresses are not directly accessible using diffraction methods, they are analyzed by measuring the associated lattice strains 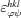. Considering equation (1), the lattice strain 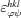 determined for an angle set (φ, ψ) with respect to the sample reference system becomes
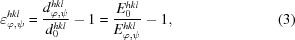
where 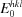 denotes the energy that corresponds to the strain-free lattice spacing 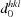. Taking into account the depth dependence of the residual strain state, the fundamental equation of XSA for the ED mode of diffraction becomes
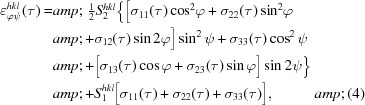
where 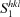 and 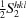 are the diffraction elastic constants (DEC). Strain data obtained by means of the 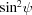 method (Macherauch & Müller, 1961 ▸) can be evaluated using two different approaches. Applying the 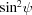 analysis to each diffraction line *E* ^*hkl*^ and plotting the corresponding stress data versus the maximum information depth 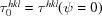 yields a first robust approximation of the residual stress depth profiles in the Laplace space, 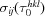 [the modified multi-wavelength method (MMWP); Klaus & Genzel, 2019 ▸]. Strain depth profiles 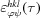 that show a high quality can be directly converted into discrete stress depth profiles σ_*ij*_(τ) by means of the universal plot (UP) method (Ruppersberg *et al.*, 1989 ▸), which results in a more detailed view of the near-surface residual stress state. Real-space depth profiles σ_*ij*_(*z*) are obtained by fitting the Laplace transforms of appropriate functions to the experimentally measured stress data. Both evaluation methods are available in *EDDIDAT*.

## 3. Hardware and software requirements   

*EDDIDAT* is based on the MATLAB programming language (toolboxes used: optimization toolbox, image toolbox, statistics toolbox). The program runs under Microsoft Windows on current systems and has been tested extensively on Windows 7 and Windows 10. The only system recommendation is a minimum screen resolution of 1920 × 1080 in order to fit all components of the graphical user interface (GUI) optimally to the screen. It is not necessary to have a MATLAB license to run the program. Only the MATLAB runtime component is required, which is delivered with the program executable.

## 4. GUI layout   

**Figure 1:**
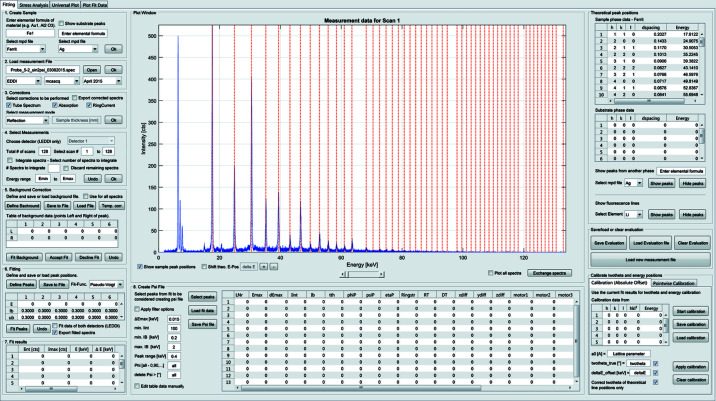
The layout of the GUI of *EDDIDAT*. The user can choose from four main tabs.

The design of the *EDDIDAT* GUI has been chosen to be user friendly and self-explanatory (see Fig. 1 ▸). Four different tabs are currently available in the GUI main window. The first tab, ‘Fitting’, is for the evaluation of EDD data loaded via input files. The second and third tabs, ‘Stress Analysis’ and ‘Universal Plot’, are for the calculation of residual stresses utilizing the fitted diffraction data. The fourth tab, ‘Plot Fit Data’, is for the graphical representation of the results. During the installation of the software, a specified folder structure is generated which serves to facilitate the administration of the internal databases, the input measurement data and the output results files.

### 4.1. The ‘Fitting’ tab   

**Figure 2:**
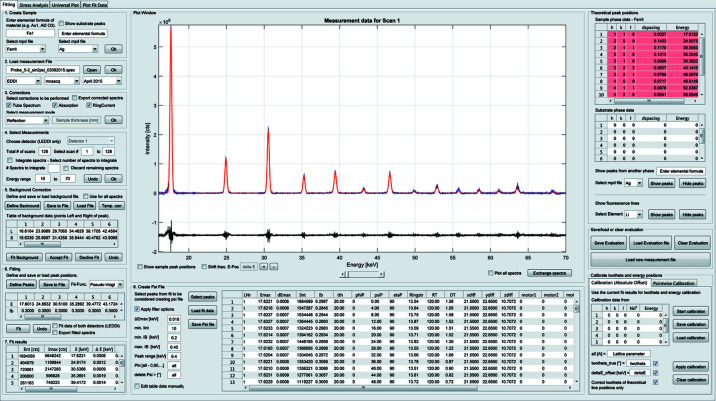
The GUI after a finished fitting process of a ferritic steel sample. The ED spectrum was measured under 2θ = 20° and 14 diffraction lines were fitted using a pseudo-Voigt function.

In the ‘Fitting’ tab (see Fig. 2 ▸) the measured ED diffraction spectra are analyzed. In order to facilitate the evaluation of the measurement data by the user, the steps of the procedure are numbered. As a first step, a virtual sample object is created in which the user enters the information about the investigated material in the form of the chemical formula. Further necessary information about the material, such as the atomic weight, material density, crystal structure and lattice parameters, is specified by selecting a corresponding material-parameter data file (mpd file) from the provided database. If a material is not available in the database, the user can easily create and add it. In the next step, the user selects the measurement to be analyzed and the instrument (LIMAX-70, LIMAX-160, LEDDI, EDDI) and detector (*e.g.* Ge, SiLi) used for the measurement. Depending on the detector used for the measurement, the appropriate correction function for the count-rate-dependent energy shift (Denks & Genzel, 2007 ▸) is applied, which is referred to in the GUI as dead-time (DT) correction.

The measured spectra are then corrected with regard to the source used, absorption and the applied measurement mode (*e.g.* reflection, transmission). Now, the user can select the energy range required for the evaluation. In addition, it is possible to integrate over a selected number of spectra before continuing with the evaluation. This is often needed in the case of measurements with very short counting times in order to improve the signal-to-noise ratio or in the case of materials with preferred orientation. In the next step, the measured spectra are corrected with regard to the background. Therefore, the user has to select points in the plot window which are then used to subtract the background. The positions of the peaks that should be analyzed are defined likewise. In the case of experiments where the temperature is changed during the measurements, strong peak shifts are likely to appear, which could affect the fitting process. In *EDDIDAT* it is possible to generate a correction function that will take into account the temperature-induced peak shift which can be applied to the user-generated background points as well as the peak positions.

In order to simplify the identification of the diffraction lines of the investigated material, all theoretical energy positions (calculated from the crystal structure information provided in the mpd file) in the selected energy range are displayed automatically in the plot window in the form of red lines. The corresponding Miller indices *hkl*, the lattice spacings *d* ^*hkl*^ and the energies *E* ^*hkl*^ are summarized in a table next to the plot window. In addition to the energy positions of the investigated material, the user can select to display the theoretical energy positions of any material from the database as well as the fluorescence lines of any element. This could be used to identify peaks in multiphase materials. The user can choose between different functions to fit the diffraction lines: Lorentzian, Gaussian, pseudo-Voigt or Thompson–Cox–Hastings pseudo-Voigt (Thompson *et al.*, 1987 ▸). In the case of strongly overlapping peaks, the peak positions can be restrained in order to prevent failure of the fitting process. The program automatically detects the number of peaks in the selected background range and fits up to six (overlapping) peaks per background range. The user-defined background and peak points can be saved to a file and reused later. The most important results of the fit procedure are summarized in a table for a first user review.

The measurement-relevant parameters and the results are summarized in a table below the plot window and can be stored in a ‘psi’ file. The user can filter the results using built-in filters (*e.g.* peak number, Δ*E* ^max^, minimum/maximum integral breadth, minimum integrated intensity, phi angle *etc*.) or manually select and delete uncertain measured data points that would negatively affect the fit procedure. The entire evaluation can be saved after each processing step, allowing later processing or sharing of results with other users. In order to analyze multiphase materials, the user has to repeat the evaluation for each phase, since the absorption correction is applied to the whole spectrum. If only the evaluation of the energy positions is desired, the user can skip the absorption correction and fit all peaks from each phase at once, and then select the respective peaks and save them to separate psi files.

In the case of residual stress measurements, it is of great importance to align the source and the detector very precisely to the diffractometer center and to adjust the height of the sample exactly. In order to check the adjustment of the instrument, a calibration measurement is carried out on a stress-free sample. On the basis of this calibration measurement, the actual 2θ angle, *i.e.* the detector position, and the absolute energy offset Δ*E* can be determined. If the adjustment shows a systematic deviation, *e.g.* because of an insufficient adjustment or as a result of the geometry of the sample, the measured energy positions can be corrected by applying a user-defined function. *EDDIDAT* provides the sub-tabs ‘Calibration’ and ‘Pointwise Calibration’ for this purpose. In the latter case, the energy positions are corrected as a function of the tilt angle ψ using up to fifth-degree polynomials.

### 4.2. The ‘Stress Analysis’ tab   

**Figure 3:**
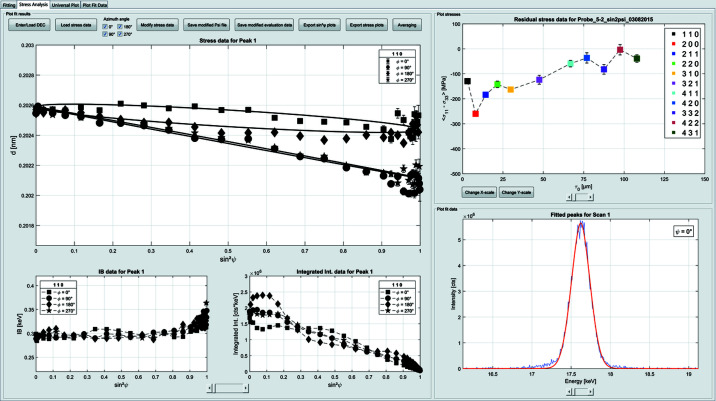
Results from the residual stress analysis on a ferritic steel sample. Measurements were performed at the azimuths φ = 0, 90, 180 and 280°. The resulting residual stress depth profile for the σ_11_ stress component shows a pronounced gradient of compressive stresses.

The stress analysis is based on the MMWP method introduced in Section 2. The DEC are required to calculate the residual stresses and have to be entered by the user. The program automatically recognizes the Miller indices *hkl* of the fitted peaks and loads the corresponding DEC from the database, if the material’s DEC are available. Otherwise, the values must be entered manually but can then be saved for further use. The actual stress analysis is initiated by activating the ‘Load stress data’ button. The program automatically recognizes the azimuthal angles (φ = 0, 90, 180, 270°) under which the measurements were recorded and determines the corresponding stress components σ_*ij*_. The resulting *d* ^*hkl*^ versus sin^2^ψ distributions and the calculated regression lines are displayed in the first plot window (see Fig. 3 ▸). Another plot window shows the resulting residual stress depth distribution(s) based on the MMWP method. In addition, plots of the integral width and integral intensity as well as plots of the fitted peaks are displayed in separate plot windows. These plots allow a graphical evaluation of the results. In the case of fitting errors, the integral width or integral intensity would lead to unusual values. In order to improve the fitting procedure, the corresponding lattice parameter values can be selected and deleted from the plot. *EDDIDAT* keeps processing the stress calculation with respect to the changes made here. As a result, the residual stress depth distribution is updated automatically (and changes are visualized). This allows the user to directly track the effect of deleting uncertain measured data points on the residual stress depth distribution. All plots shown in this tab can be saved as TIFF graphics and ASCII files. In the case of the stress plots, a tau file is generated to store the values of the stress-free lattice parameters 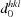 and the stress components 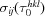.

### 4.3. The ‘Universal Plot’ tab   

**Figure 4:**
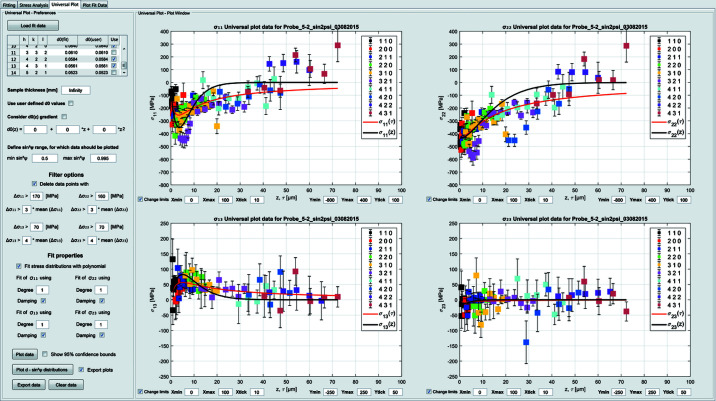
Results from the residual stress analysis on a ferritic steel sample using the UP method. The Laplace (red) and the real-space (black) residual stress depth profiles are shown as lines.

**Figure 5:**
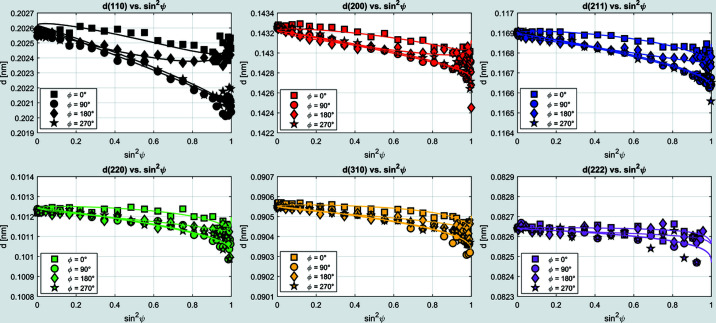
Re-calculated *d* ^*hkl*^–sin^2^ψ distributions of the first six diffraction lines using the fitted residual stress depth function from the UP evaluation.

The stress analysis is based on the UP method introduced in Section 2. There is a plot window for each of the four stress components (see Fig. 4 ▸). The evaluation requires the user to have already processed the measurement data using the ‘Stress Analysis’ tab. The necessary peak information is shown using the button ‘Load fit data’. The resulting table allows the user to select the peaks to be considered for the analysis. The user can define the sample thickness (*e.g.* this is important in the case of thin films) and can decide whether the 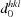 values from the previous fit with the ‘Stress Analysis’ tab or predefined 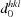 values should be used for the stress evaluation. Furthermore, it is possible to account for a 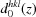 gradient in the sample using a polynomial with up to three parameters. In order to prepare the data for fitting, the user can select the sin^2^ψ range for which data should be plotted and can delete data points using various filters. The data can be plotted without fitting, such that the user can judge the quality of the data preparation. The residual stress depth profiles can then be fitted using simple or exponentially damped polynomials up to degree five. To assess the quality of the fitted residual stress depth distributions it is possible to recalculate *d* ^*hkl*^–sin^2^ψ curves by inserting the fitted σ_*ij*_(τ) progressions into equation (4) and to compare them with the measured *d* ^*hkl*^–sin^2^ψ distributions (see Fig. 5 ▸). All plots shown in this tab can be saved as TIFF graphics and ASCII files.

### 4.4. The ‘Plot Fit Data’ tab   

**Figure 6:**
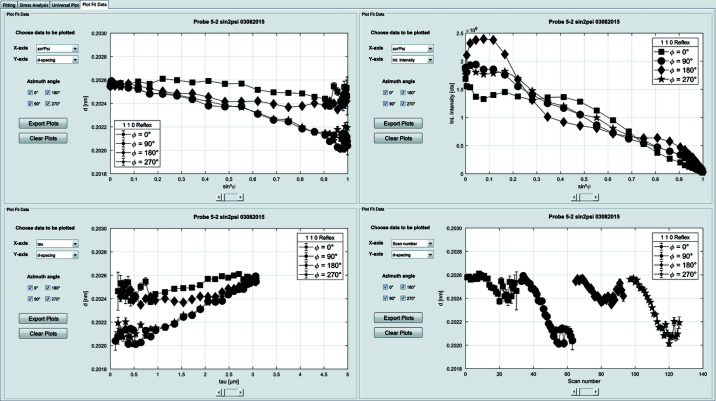
The ‘Plot Fit Data’ tab can be used to assess and compare different peak parameters as a function of multiple variables.

In this tab (see Fig. 6 ▸) the results of the analysis can be plotted in four separate graphs. The determined line-profile properties (energy position, lattice parameter, integral width, integral intensity *etc*.) can be plotted as a function of a multitude of parameters (ψ, sin^2^ψ, information depth τ, temperature *T*, scan number *etc*.), and can be exported as TIFF graphics and ASCII files.

## 5. Outlook   

For future versions of *EDDIDAT*, we are planning to extend the program by adding an advanced evaluation approach (Apel *et al.*, 2014 ▸) based on the Rietveld method (Rietveld, 1967 ▸), in order to calculate real-space residual stress depth profiles σ_*ij*_(*z*). In addition to stress analysis, many users are interested in the (depth-resolved) analysis of the microstructure. For this purpose, we plan to implement data analysis based on the Rietveld method following Apel *et al.* (2011 ▸) and further line-profile analysis methods such as the Williamson–Hall plot method (Williamson & Hall, 1953 ▸). It would then be possible to perform a line-profile analysis on ED data. With regard to quantitative phase analysis, it is planned to implement a search-and-match routine. Additionally, the program is designed to analyze measurements from different sources (synchrotron, MetalJet, conventional X-ray tubes *etc*.), allowing one to add new devices and their features in order to evaluate EDD data measured on those devices. Currently, this can only be carried out manually by the authors as requested by users. For future versions we will implement a method for the users to add new devices themselves.

## 6. Distribution   

*EDDIDAT* and the corresponding documentation are freely available for academic users and can be obtained upon request from the authors. Feedback from users is welcome and can be submitted to the authors by email.
